# Screening of women with aesthetic prostheses in dedicated sessions of a population-based breast cancer screening programme

**DOI:** 10.1007/s11547-021-01357-5

**Published:** 2021-05-05

**Authors:** Silvia Deandrea, Laura Cavazzana, Niccolò Principi, Ester Luconi, Mauro Campoleoni, Anan Judina Bastiampillai, Lucia Bracchi, Lauro Bucchi, Stella Pedilarco, Antonio Piscitelli, Maria Silvia Sfondrini, Anna Rita Silvestri, Silvana Castaldi

**Affiliations:** 1 Health Protection Agency Metropolitan City of Milan, Milan, Italy; 2 Post Graduate School of Public Health, University of Milan, Milan, Italy; 3 Fondazione IRCCS Ca’ Granda Ospedale Maggiore Policlinico, Milan, Italy; 4 Medical Physics Unit, Fondazione IRCCS Ca’ Granda Ospedale Maggiore Policlinico, Milan, Italy; 5 Romagna Cancer Registry, Romagna Cancer Institute (IRCCS Istituto Romagnolo per lo Studio dei Tumori, IRST, “Dino Amadori”), Meldola, Forlì, Italy; 6 Division of Radiology, Fondazione IRCCS Ca’ Granda Ospedale Maggiore Policlinico, Milan, Italy; 7 Department of Biomedical Sciences for Health, University of Milan, Milan, Italy; 8 Quality Unit, Fondazione IRCCS Ca’ Granda Ospedale Maggiore Policlinico, Milan, Italy

**Keywords:** Breast implants, Breast neoplasms, Diagnostic screening programmes, Mammography

## Abstract

**Background:**

Women with aesthetic prostheses must be included in the target population of mammography screening programmes. Breast implants are radiopaque and partially obscure the breast tissue. This can be avoided with the use of the Eklund technique, which causes an increased radiation exposure. In this study, augmented women undergoing a dedicated protocol within a population-based screening programme were compared according to selected indicators with the standard screening population. Essential dosimetric parameters and their time trend were also assessed.

**Materials and methods:**

The study was conducted in a screening centre in Milan in the years 2009–2016. The screening protocol for women with breast implants included a double-read mammography with the Eklund views, ultrasound and clinical breast examination.

**Results:**

A total of 28,794 women were enrolled, including 588 (2%) women with breast implants and 28,206 (98%) undergoing the standard screening protocol. The invasive assessment rate was 9.0‰ for women with breast implants vs. 15‰ in the standard cohort. The surgical referral rate was 2.2% vs. 0.9%. The detection rate was similar in the two groups (4.0 and 4.5‰, respectively). There were significant differences in the average glandular dose according to the mammography equipment. The use of the Eklund views increased over time.

**Conclusions:**

Screening of augmented women according to a specific protocol in the contexts of population-based programmes is feasible. Observed differences in screening indicators relative to the standard screening population require further research. The increasing use of Eklund views probably results from quality assurance measures associated with screening programmes.

## Introduction

Breast augmentation is one of the most frequently performed aesthetic surgeries among women. Approximately, 5 to 10 million women worldwide have breast implants [1]. The American Society of Aesthetic Plastic Surgeons has reported an increase of 207% from 1997 to 2016, showing an exponential growth in recent years [2]. In Italy, according to data for 2017, nearly 50,000 breast augmentation surgeries are done annually [3].

Over the last years, the scientific community has raised the hypothesis that breast implants might be related to an increase in the incidence of breast cancer. In fact, many studies [4–6] have shown that implants have no effects on the risk of disease, although an increase in the incidence of the rare implant-associated anaplastic large cell lymphoma has been reported [7].

A different concern has arisen from the fact that breast implants are radiopaque and, in part, obscure the breast tissue. Some studies have highlighted that the prosthesis and its location could make early cancer detection by mammography more challenging, eventually leading to delay in diagnosis [8–16]. In spite of that, mammography remains an accurate method of early detection of breast cancer in women with prostheses [17, 18]. For this reason, the 2006 European guidelines for quality assurance in breast cancer screening and diagnosis recommend that breast cancer screening programmes (BSP) offer mammography to all women in the target age range, including those with aesthetic prosthesis [19]. Notably, however, the European guidelines recommended that these women should be screened in clinics where ultrasound (US) is available and that radiographers should receive specific training.

Indeed, the physical presence of an implant may hamper the application of standard radiographic procedures and stresses the need for a specific management protocol, including special views with back placement of the implant (Eklund views), and a specific technical expertise of the radiographer [20]. Eklund views are performed by displacing the implant posteriorly against the chest wall, pulling the breast tissue over and in front of the implant. This technique allows better visualization of the glandular tissue, in particular for craniocaudal views [21, 22]. On the other hand, when additional views are needed in order to perform the Eklund technique, women are exposed to a greater dose of radiation [11, 23]. The factors that affect the absorbed radiation dose can be summarized in the thickness of the tissue analysed, the amount of compression used, and the number of views taken.

In 2009, in order to provide women with aesthetic breast prostheses with an appropriate management within the local population-based BSP, the Milan Local Health Authority implemented a specific screening protocol in a reference centre, operating in one of the screening centres. This study is the first Italian study to describe the management of these women within a population-based BSP. Its objective is to report (1) the main screening and management indicators in comparison with nonaugmented women undergoing standard screening in the same period, and (2) the main dosimetric parameters and their trend over time.

## Materials and methods

### Setting

In the city of Milan (about 1.300.000 inhabitants), the BSP for women aged 50–69 (approximately 200,000 for each screening round) was started in 1999–2000. The process to extend the target age to 45–74 was undertaken in 2016, although women aged 45–49 and 70–74 had already been screened upon request. The interval is biennial for women aged 50–74 and annual for women aged 45–49. Standard indicators are regularly provided to the Department of Health of the Lombardy regional Administration [24] and the National Centre for Screening Monitoring [25]. The average response rate is 67% and the recall rate is 10% for the first screens and 4% for subsequent ones.

Between 2009 and 2016, the reference centre for the screening of women with breast implants has been at the Breast Radiology Unit of the Fondazione IRCCS Ca’ Granda Ospedale Maggiore Policlinico (henceforth “Policlinico”), a public teaching and research hospital located in the centre of Milan. The present study focuses on this period. From 2017 onwards, due to a re-organization of healthcare services following the Lombardy Region healthcare reform [26], screening women with breast implants is no longer the responsibility of the BSP, although the Unit still provide early diagnosis for physician-referred and self-referred women with breast implants. The Breast Radiology Unit of the Policlinico is a hub for the diagnosis and treatment of breast diseases (with about 33,000 exams per year). It performs approximately 12% of screening mammographies and further assessment tests for the BSP covering the area of Milan [27, 28].

### Standard screening protocol

During the study period, all women eligible for the BSP of the Milan Local Health Authority received a personal appointment letter. Self-referral of eligible women was also accepted. The screening mammography and the diagnostic assessment tests were provided by centres established in hospital-based radiology services. All women had a two-view digital mammography with independent double reading. Selected early recall (assessment after 6 months) and early rescreening (screening mammography after 1 year) policies were practiced. Women with positive results (BI-RADS 3–5) were contacted by telephone and referred for diagnostic assessment, which included supplementary mammography, US, clinical breast examination (CBE), and cytological and histological invasive procedures—if appropriate.

### Screening of women with breast prostheses

The screening invitation letters included a sentence asking women with aesthetic prostheses to contact the BSP communication centre. For women adhering to this recommendation, a specialized appointment was set in the Breast Radiology Unit of the Policlinico. The Unit was responsible both for the screening of women and their diagnostic work-up. These were done in dedicated screening sessions (also referred to as *prosthesis pathway*) and included a mammography performed by a trained technician and double-read, with the Eklund technique in the craniocaudal views, and US and CBE performed by a radiologist. In the case of a negative result, women were re-invited at 1-year or 2-year intervals depending on the clinical opinion. In the case of a suspicious result, women underwent the same assessment tests as those offered to standard screenees.

### Mammography systems

Up to January 2015, women were screened with two first-generation full field digital mammography systems, i.e. a General Electric Senograph 2000D (henceforth “2000D”) and a General Electric Senograph Essential (henceforth “ES”). From January 2015 on, women have been screened with a more recent Hologic Selenia Dimensions. This new equipment is provided with a specific post-processing software which improves the image quality in the presence of an implant. All equipments underwent a quality control programme for the assessment of the physical and technical requirements according to the EUREF protocols [29].

### Study population

This is a retrospective cohort study. The cohort included all carriers of sub-glandular or retro-glandular aesthetic breast implants who entered the prosthesis pathway between November 2009 and July 2016.

The cohort of women undergoing the standard protocol of the BSP at the Policlinico during the same time period was used as a control group. Inclusion criteria were female sex, age between 45 and 74, and no previous history of breast cancer.

### Data collection

For women undergoing the standard protocol of the BSP, data on basic screening and assessment of positive findings were real-time recorded during the daily operation. Histological information for screen-detected lesions was collected via search in the regional archive of the Hospital Discharge Records and in the databases of Pathology Departments at the surgical referral hospitals.

For the cohort of women with breast implants, information was collected from the Cancer Registry of the Agency for Health Protection of Milan (formerly, Milan Local Health Authority). A dataset was created that included demographic variables (age, nationality and residence), screening history, screening and assessment tests (type, date, results), and screening episode result.

The collection of dosimetric parameters was performed on a representative sample of the total number of images (47.6%). For each image, the following data were collected: average glandular dose (AGD), breast thickness (BT), Eklund’s implant-displacement technique (yes/no) and exposure parameter (kV, mAs, anode-filter combination, and compression force).

### Definitions

In this article, screening episode indicates each single participation by all women to the biennial screening. The term session indicates a single time slot allocated for women within the screening episode. Screening round (first, subsequent) indicates the first participation and the subsequent ones.

### Data analysis

Women with and without breast implants were compared according to age, year of mammography, nationality, screening round, and number of screening episodes using the chi-square test. Age was classified in five-year intervals and the year of mammography in two-year intervals. Univariate logistic regression models were built to test for the presence of a significant association between the presence of a breast implant and the age class (reference, 48–49), year of mammography (reference, 2009–2010), and nationality (reference, other, i.e. not Italian).

Assessment tests were further classified into non-invasive (mammography, CBE, US, and MRI) and invasive (needle core biopsy (NCB) and fine needle aspiration (FNA)). Rates, and 95% confidence intervals (CI), were computed for invasive assessments, surgical referral (including both surgical biopsy and surgical intervention), early recall/rescreen and detection of cancer (or detection rate (DR) per 1000 women). Data are presented for women with breast implants and for the standard screening population, which considers all women screened and those referred for assessment separately. Due to the possibility of sending augmented women to a 1-year appointment, the 6-month recall was considered for the early recall/rescreen rate computation for the standard screening cohorts only.

Women with breast implants who underwent the whole dedicated screening procedure (mammography, US and CBE) were defined as screened per protocol. With the aim of disentangling the effect of a “per protocol” procedure and that of being a prosthesis carrier, we matched cases (augmented women) and controls from the standard screening population according to age interval, screening round and per-protocol status. The odds ratio (OR) for surgical referral was calculated from the contingency table (breast implant and surgical referral). For invasive examinations, the analysis was not performed, as there were no “per protocol” matched women without breast implants.

In order to determine whether the sample subjects with dosimetric information differed from those with no information in terms of distribution by age class, year of mammography, nationality and screening round, a two-sided Fisher exact test was performed. The level of statistical significance was set at *P* < 0.05. The sample was demonstrated to be representative of the population of women with breast implants.

For all the views (right craniocaudal, RCC, left craniocaudal, LCC, right mediolateral oblique, RMLO and left mediolateral oblique, LMLO), the mean and median AGD and BT and their range were calculated for the whole sample, for different mammography systems (2000D vs. ES) and for the application of the Eklund technique (yes vs. no). T tests were used to evaluate the significance of the difference between mean AGDs. Each view was independently considered.

Finally, the significance of the increasing use of the Eklund procedures over time, using the semester as a time unit, was tested with an Armitage test versus the null hypothesis on no linear trend.

The management of data sets was done with the program KNIME Analytics Platform ver. 3.6.2 [30]. Statistical analyses were performed using the program “R” ver.3.5.1 (2018) [31].

## Results

### Patient characteristics

Between November 2009 and July 2016, 28,794 women participated in the BSP at the Breast Radiology Unit of the Policlinico. As shown in the upper row of Table 1, there were 588 (2%) women with breast prostheses and 28,206 (98%) women who were screened according to the standard protocol.

**Table 1:** Women’s demographic and clinical characteristics at first observation in the study

Characteristics	Women with breast implants n (%)	Women without breast implants n (%)	*χ*^2^test (likelihood)^a^
Number	588 (2.0)	28,206 (98.0)	
Age class			
48–49	5 (0.9)	151 (0.5)	*p* < 0.001
50–54	272 (46.3)	6479 (23.0)	
55–59	167 (28.4)	4478 (15.9)	
60–64	69 (11.7)	5317 (18.9)	
65–69	58 (9.9)	6177 (21.9)	
70–74	17 (2.9)	5604 (19.9)	
Mean age	57.15 (sd 5,10; range 49–74)	62.16 (ds 7,03; range 48–74)	
Year of mammography			* p* < 0.001
2009–2010	60 (10.2)	9560 (33.9)	
2011–2012	249 (42.4)	9906 (35.1)	
2013–2014	262 (44.6)	4210 (14.9)	
2015–2016	17 (2.9)	4530 (16.1)	
Nationality	*p* = 0.351		
Italian	530 (90.1)	25,086 (88.9)	
Other	58 (9.9)	3120 (11.1)	
Screening round			*p* = 0.764^b^
First	197 (33.5)	9284 (32.9)	
Subsequent	391 (66.5)	18,922 (67.1)	
Number of screening episodes			*p* < 0.001^c^
1	278 (47.3)	9980 (35.4)	
2	229 (39.0)	6001 (21.3)	
3	68 (11.6)	9273 (32.9)	
≥ 4	13 (2.2)	2952 (10.5)	
Number of screen-detected cancers	4	274	-

^a^*p* < 0.05 from *χ*^2^ test identifies a significant association between the explanatory variable (for example, age) and the response variable (breast implant or not breast implant). In particular, the *X*^2^ from a likelihood compares the model only with an intercept and the model with the intercept and the variable

^b^the logistic regression for the chi square test was codified with the response variable as 1 in case of subsequent screening round and as 0 otherwise. The explanatory variable was codified as 1 in the presence of a breast implant and 0 otherwise

^c^Chi-square test: *p* < 0.05 H_0_: there is not an association between the presence of breast implant and the number of screening episodes. Ha: there is an association between the presence of breast implant and the number of screening episodes

Table 1 shows the distribution of women in both cohorts according to age, year of mammography, nationality, screening round, and number of screening episodes, as well as the number of breast cancers detected. There was evidence for an inverse association between the age class and being a breast implant carrier. Augmented women had more often a single screening episode. Their different distribution by year of mammography reflected the gradual roll-out of the dedicated screening programme. Nationality and the number of screening rounds were not associated with the odds of a woman having a breast implant.

The OR from multiple regression analysis, shown in Table 2, was 1.44 (95% C.I.: 1.16–1.79) for the age class 65–69, and 1.81 (95% C.I.: 1.47–2.25) for the age class 70–74 as compared with the class 48–54. For the remaining classes, the OR was not significantly different from the unity.

**Table 2:** Odds Ratios from multiple regression analysis for age classes compared with the class 48–54

Characteristic		Odds Ratio (95% confidence interval)
Age class	< 55	Reference
	55–59	1.07 (0.85—1.35)
	60–64	1.20 (0.94—1.53)
	65–69	1.44 (1.16—1.79)
	70–74	1.81 (1.47–2.25)

### Screening and management indicators

As shown in the upper row of Table 3, the total number of screening episodes was 67,991, including 61,608 screening mammography sessions, 5384 assessment sessions and 999 prostheses protocol sessions. Table 3 also gives the number of tests and the indicators for women with breast implants, for women without breast implant (standard population) and for women without breast implant undergoing assessment only. Most women (87.3%) were screened as per protocol, whilst for others one or more of the three tests of the protocol were not performed. Invasive assessment rate was 9.0‰ for women with breast implants. The corresponding figure for women undergoing screening mammography was 15.2‰, and for women undergoing assessment 174.8‰. Surgical referral rate was 2.2% for women with breast implants, 0.9% for women undergoing screening mammography and 9.7% for women undergoing assessment. Finally, the DR was 4.0‰ in the augmented group and 4.5‰ in the control group.

**Table 3:** Screening and management indicators for women with breast implants and women without breast implant (all women with a screening mammogram and women undergoing assessment only)

Indicator	Women with breast implants	Standard screening population	
		Women without breast implants—all	Women without breast implants—assessment only
Screening episodes (*n*)	999	61,608	5384
Mammography (*n*, %)	956 (95.7)	4269 (6.4)	4269 (79.3)
CBE (n, %)	915 (91.6)	4689 (7.0)	4689 (87.1)
US (n, %)	965 (96.6)	4831 (7.2)	4831 (89.7)
MRI (n, %)	2 (0.2)	49 (0.1)	49 (0.9)
FNA (n, %)	0 (0.0)	33 (0.1)	33 (0.6)
NCB-VAB (n, %)	9 (0.9)	867 (1.3)	867 (16.1)
Invasive assessment, rate (‰, 95% CIs)			
FNA	0	0.5 (0.4–0.8)	6.1 (4.2–8.6)
NCB-VAB	9.0 (4.1–17.0)	14.7 (13.8–15.7)	168.7 (158.7–178.9)
Total	9.0 (4.1–17.0)	15.2 (14.2–16.5)	174.8 (162.9–187.5)
Surgical referral rate (%, 95% CIs)	2.2 (1.4–3.3)	0.9 (0.8–0.9)	9.7 (9.0–10.6)
Early recall/rescreen rate (%, 95% CIs)	2.4 (1.4–3.3)	0.9 (0.8–1.0)	10.0 (9.2–10.8)
Detection rate (‰, 95% CIs)	4.0 (1.1–10.2)	4.5 (3.9–5.0)	-

*FNA*, Fine needle aspiration;  *NCB-VAB*, Needle Core Biopsy/Vacuum Assisted Biopsy; *CI*, Confidence Interval

When matched with nonaugmented women according to age interval, screening round and non-invasive tests performed as “per protocol”, breast implant carriers showed a reduced risk to be referred to surgery (OR, 0.25; 95% CI, 0.14–0.43), suggesting a different management of these women not attributable to demographics or screening history characteristics.

### Dosimetric parameters

In Table 4, the mean and the median for AGD and for BT for the whole sample, for different equipments and for the application of the Eklund technique are reported. As expected, the Eklund technique was usually not performed in MLO views, and compression was higher with the Eklund displacement. We observed a significantly (*p* < 0.001) lower AGD with the Selenia system vs. 2000D and ES for all the four views.

**Table 4:** Average glandular dose (AGD) and for BT (breast thickness) for the four views

	RCC		LCC		RMLO		LMLO	
	AGD (mGy)	BT (mm)	AGD (mGy)	BT (mm)	AGD (mGy)	BT (mm)	AGD (mGy)	BT (mm)
**Total**								
Views	452	473	450	469	397	473	390	469
Mean	1.4	48.8	1.4	47.6	1.2	69	1.2	67.8
Median	1.4	45.0	1.4	45.0	1.2	71	1.2	69
Range	0.6–7.7	18–108	0.5–2.7	20–127	0.1–3.9	29–110	0.7–2.7	31–118
**Systems**								
*2000D/ES*								
Views	381	402	382	401	326	402	322	401
Mean	1.5	48.8	1.5	47.9	1.3	67.2	1.3	66.1
Median	1.4	45	1.5	45	1.2	69	1.2	67
Range	0.7–7.7	18–105	0.7–2.7	20–127	0.6–2.2	29–110	0.7–2.7	31–118
*Selenia*								
Views	71	71	68	68	71	71	68	68
Mean	1.1	48.6	0.9	46.1	1.1	78.9	1.1	78.3
Median	0.9	43	0.8	41	1	78	1	77
Range	0.6–6.2	21–108	0.5–2	23–100	0.1–3.9	53–106	0.8–1.6	44–110
**Eklund**								
*Yes*								
Views	339	345	347	357	13	14	5	5
Mean	1.4	43.3	1.4	42.6	1.5	47.1	1.6	47.8
Median	1.5	45	1.5	45	1.5	45	1.4	45
Range	0.6–4.8	18–80	0.5–2.7	20–75	1.1–2.2	29–77	1.3–2.4	31–66
*No*								
Views	113	128	102	111	384	459	385	464
Mean	1.3	63.5	1.2	63.1	1.2	69.6	1.2	68.1
Median	1.1	59	1.2	55	1.2	71	1.2	69
Range	0.8–7.7	37–108	0.7–2.7	31–127	0.1–3.9	41–110	0.7–2.7	31–118
**2000D/ES vs. Selenia**								
*t* test	− 3.88	–		− 14.04			− 3.55	
df	80.01			93.25	–		87.11	–
*p* value	< 0.001			*p* < 0.001			< 0.001	
95% CI	− 0.57 to 0.18			− 0.70 to 0.53			0.28 to 0.28	

RCC, right craniocaudal; LCC, left craniocaudal; RMLO, right medial–lateral oblique; LMLO, left medial–lateral oblique

### Dosimetric parameters by Eklund technique

In Table 5, AGD and BT for the different equipment are reported separately for Eklund technique (yes vs. No). When the technique was performed, AGD was confirmed to be lower with an automatic exposure control or an optimized exposure protocol (Selenia).

**Table 5:** Average glandular dose (AGD) and for BT (breast thickness) for the four views according to different machines and Eklund technique

2000/ED						Selenia					
		RCC		LCC				RCC		LCC	
Eklund (mm)		AGD (mGy)	BT (mm)	AGD (mGy)	BT (mm)	Eklund		AGD (mGy)	BT (mm)	AGD (mGy)	BT
Yes	Mean	1.5	43.7	1.6	43	Yes	Mean	1	41.2	0.8	40
	Median	1.5	45	1.6	45		Median	0.9	40	0.7	39
	Range	0.7–4.8	18–80	0.7–2.7	20–75		Range	0.6–4	21–68	0.5–2	23–70
No	Mean	1.3	61.6	1.3	61.2	No	Mean	1.4	79	1	81
	Median	1.2	56	1.2	54		Median	1	79	0.9	90
	Range	0.8–7.7	37–105	0.7–2.7	31–127		Range	0.8–6.2	43–108	0.9–1.6	44–100

RCC, Right Craniocaudal; LCC, Left Craniocaudal; RMLO, Right Medial–Lateral Oblique; LMLO, Left Medial–Lateral Oblique

### Prevalence of the Eklund technique over time

The prevalence for the Eklund technique over different periods was determined. There was a significant linear trend toward an increased implementation of the technique, from 45% in the first semester of activity to 83% in the last one (Armitage test, chi-squared test for trend, 43.59; degrees of freedom, 1; *p* value < 0.001) [data not shown].

## Discussion

### Principal findings

The indicators studied provided evidence for a different management of augmented women compared with women undergoing the standard basic screening. Albeit similar to women in the screening cohort with respect to the risk of breast cancer, augmented women underwent the same tests as women referred for assessment because of suspicious mammograms. For this reason, neither of the two cohorts is an appropriate comparator for the augmented cohort. By implication, our results should mainly be used for healthcare planning purposes, that is, for assessing the potential workload and the investment needed in breast care services.

From this standpoint, our data suggest that augmented women screened according to this protocol do not require a higher volume of invasive tests than the standard screening population. The surgical referral rate, conversely, was higher. However, there was an inverse association between surgical referral and being a prosthesis carrier when women were matched with subjects undergoing the same protocol (mammogram + US + CBE). This result suggests a different management possibly according to clinical suspicion, to setting preference (inpatient surgical biopsy vs. outpatient), and to some unmeasured factors related to the status of prosthesis carrier.

The second key finding of the study is that the dosimetric parameters changed over time due to the upgrade to a more advanced technology (Selenia). In addition, radiographers have improved their capability in applying the Eklund technique as a first choice method for the craniocaudal views. This observation must be interpreted considering the advantage in terms of resources, staff education and professional improvement when cases requiring special skills are concentrated in a high-volume centre.

### Rationale issues

The rationale of setting-up sessions of BSPs specifically dedicated to women with breast implants relies on the 2006 European guidelines for quality assurance in breast cancer screening and diagnosis. These, however, recommend that mammographic imaging be performed in clinics where US is available and, more important, that radiographers be specifically trained and have up to date information and knowledge about breast implants [19]. It must be carefully considered that training and quality assurance are priorities in the implementation of BSPs.

Regarding the rationale for this study, two points need to be made. Firstly, the publication of our data was justified by the fact that the results of protocols for the management of women with breast implants attending BSP in Italy and elsewhere have never been reported in relevant publications.

Secondly, there is a general consensus that that a mammogram is of high quality when it enables the radiologist to discern the presence or absence of the mammographic features of breast cancer in the image with high sensitivity and specificity [32], and when it yields adequate diagnostic information with the least possible radiation exposure to the breast [33, 34]. These requirements are particularly needed when screening women with breast implants. This is the reason why the endpoints of this study encompassed both the screening and management indicators and some essential dosimetric parameters.

### Comparison with the literature

We are not aware of previous publications reporting data comparable with ours. However, protocols for the management of women with breast implants in the screening setting are operating in other countries including, to our knowledge, Australia [35], UK [17] and Spain (Cantabric regional programme) [36]. All three protocols include dedicating additional time to these women and performing mammography in dedicated screening slots. Additional time is required because of the need to collect an accurate anamnesis about the type of prosthesis and its state [35] and to perform all required manoeuvres [17, 35]. The Spanish and the British protocols also point out that the mammogram is not aimed at assessing the state of the prosthesis, but just at early detection of breast cancer [17, 36]. The UK protocol emphasizes the radiographers’ training needs. In the NHS Breast Screening Programme (NHSBSP), indeed, all radiographers undertaking the Eklund technique should give proof that they have been educated and trained to perform the manoeuvre [17]. None of the protocols mentioned above include performing further tests, apart from those already needed for the work-up of suspicious mammograms.

What our protocol has in common with the models from Australia, UK, and Spain is that it reserves dedicated slots for women with breast implants and that the radiographic staff are trained in the Eklund technique. Also the American Cancer Society, too, highlights the importance of these practices, in order to guarantee a correct execution of the implant displacement views [37].

### Study limitations

This study had two major limitations that need to be corrected in future investigations. Firstly, our protocol is the only one offering additional non-invasive tests (US and particularly CBE), in order to overcome any detection impairment due to the prosthesis. Because of its poor statistical power, however, the study was not designed to assess the usefulness of adding other tests to mammography in order to increase the detection rate, nor to show whether these actions lead to unintended harms.

Secondly, prostheses implanted in Europe are usually silicone gel-filled and textured [3] and, in most cases, their location is partially submuscular [38]. A recent survey has shown that, elsewhere, the most common materials and locations may differ from to the ones observed in our cohort. Consequently, our results cannot be generalized to settings where prostheses have different characteristics.

In addition, we specify that the collection of dosimetric parameters was performed only on a representative sample, not the total number of images, for reason of feasibility.

## Conclusions

To our knowledge, this study is the first to report screening indicators and dosimetric parameters collected in sessions of a BSP specifically dedicated to augmented women in Italy and elsewhere. The ever-increasing prevalence of this condition will set a challenge to BSPs for the next future, both from the technical and the diagnostic perspective. In this respect, our work could represent a starting point to plan the necessary resources, when integrating screening programs for augmented women.

Despite the limitations of this study, our experience shows that the management of these patients is feasible and can improve over time—provided that they are referred to a specialized centre where the staff is properly trained and the mammography system is up-to-date and subjected to quality control programme.

## Data Availability

Data available on request from the authors.

## Abbreviations

AGD: Average glandular dose
BI-RADS: Breast Imaging-Reporting and Data System
BSP: Breast cancer screening programme
BT: Breast thickness
CBE: Clinical breast examination
CI: Confidence interval
DR: Detection rate
EUREF: European Reference Organisation for Quality Assured Breast Screening and Diagnostic Services
FNA: Fine needle aspiration
kV: Kilovoltage
LCC: Left craniocaudal
LMLO: Left mediolateral oblique
mAS: Milliamperesecond
NCB: Needle core biopsy
OR: Odds ratio
RCC: Right craniocaudal
RMLO: Right mediolateral oblique
US: Ultrasound

## Code availability

Code available on request from the authors.
